# Prognostic significance of nutritional markers in metastatic gastric and esophageal adenocarcinoma

**DOI:** 10.1002/cam4.3604

**Published:** 2020-12-09

**Authors:** Lucy X. Ma, Kirsty Taylor, Osvaldo Espin‐Garcia, Reut Anconina, Chihiro Suzuki, Michael J. Allen, Marta Honorio, Yvonne Bach, Frances Allison, Eric X. Chen, Savtaj Brar, Carol J. Swallow, Jonathan Yeung, Gail E. Darling, Rebecca Wong, Sangeetha N. Kalimuthu, Raymond W. Jang, Patrick Veit‐Haibach, Elena Elimova

**Affiliations:** ^1^ Department of Medical Oncology Princess Margaret Cancer Centre University Health Network University of Toronto Toronto ON Canada; ^2^ Department of Biostatistics Princess Margaret Cancer Centre University Health Network University of Toronto Toronto ON Canada; ^3^ Joint Department of Medical Imaging Toronto General Hospital University Health Network University of Toronto Toronto ON Canada; ^4^ Division of Thoracic Surgery Department of Surgery Toronto General Hospital University Health Network University of Toronto Toronto ON Canada; ^5^ Department of Surgical Oncology Princess Margaret Cancer Centre University Health Network and Sinai Health System University of Toronto Toronto ON Canada; ^6^ Department of Radiation Oncology Princess Margaret Cancer Centre University Health Network University of Toronto Toronto ON Canada; ^7^ Department of Pathology Laboratory Medicine Program University Health Network University of Toronto Toronto ON Canada

**Keywords:** esophageal cancer, gastric cancer, malnutrition, prognosis, sarcopenia

## Abstract

**Background:**

Malnutrition and sarcopenia are poor prognostic factors in many cancers. Studies in gastric and esophageal (GE) cancer have focused on curative intent patients. This study aims to evaluate the prognostic utility of malnutrition and sarcopenia in de novo metastatic GE adenocarcinoma.

**Methods:**

Patients with de novo metastatic GE adenocarcinoma seen at the Princess Margaret Cancer Centre from 2010 to 2016 with an available pre‐treatment abdominal computed tomography (CT) were included. Malnutrition was defined as nutritional risk index (NRI) <97.5. Skeletal muscle index (SMI) was measured at the L3 level (sarcopenia defined as SMI <34.4 cm^2^/m^2^ in women and <45.4 cm^2^/m^2^ in men). Patients receiving chemotherapy had NRI and SMI recalculated at the time of first restaging CT.

**Results:**

Of 175 consecutive patients, 33% were malnourished and 39% were sarcopenic at baseline. Patients with pretreatment malnourishment had significantly shorter overall survival (OS; 5.8 vs. 10.9 months, *p* = 0.000475). Patients who became malnourished during chemotherapy had worse OS compared to those who maintained their nutrition (12.2 vs. 17.5 months *p* = 0.0484). On univariable analysis, ECOG (*p* < 0.001), number of metastatic sites (*p* = 0.029) and NRI (*p* < 0.001) were significant prognostic factors while BMI (*p* = 0.57) and sarcopenia (*p* = 0.19) were not. On multivariable analysis, ECOG (*p* < 0.001), baseline NRI (*p* = 0.025), and change in NRI during treatment (*p* < 0.001) were significant poor prognostic factors for OS.

**Conclusions:**

In de novo metastatic GE adenocarcinoma patients, ECOG, pretreatment NRI and change in NRI were significant prognostic factors for OS while sarcopenia was not. Use of NRI at baseline and during treatment can provide useful prognostic information.

## Graphical abstract

In de novo metastatic gastric and esophageal adenocarcinoma patients, ECOG, pre‐treatment nutritional risk index (NRI) and change in NRI during treatment were significant prognostic factors for overall survival while sarcopenia was not. Use of NRI at baseline and during treatment can provide useful prognostic information.

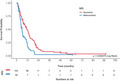

## 1 INTRODUCTION

Gastric and esophageal cancers are a major health burden worldwide.^1^ Many patients present with advanced disease, and patients are often malnourished at presentation due to disease‐related symptoms of anorexia, nausea, and dysphagia.

There is increasing interest in the prognostic significance of malnutrition in cancer patients. With the high prevalence of obesity in many populations including North America, traditional measures such as weight and body mass index (BMI) may not be ideal as screening tools for malnutrition. The Nutrition Risk Index (NRI) was developed by Buzby et al. as a simple tool to assess nutritional status, and is calculated using a patient's weight and serum albumin.^2^ In esophageal cancer, studies in patients undergoing curative intent chemoradiation or surgery have shown that both the NRI and the modified geriatric NRI (GNRI) are independent prognostic factors for survival.^3^, ^4^, ^5^, ^6^, ^7^

Another marker of poor nutrition and cachexia is sarcopenia, or the loss of skeletal muscle mass. Sarcopenia has been associated with poor outcomes in several solid tumor malignancies.^8^, ^9^, ^10^, ^11^ In recent years, several groups have investigated the prognostic effect of sarcopenia in gastric and esophageal cancers. The vast majority of these studies focused on patients with localized disease undergoing curative‐intent treatment.^12^, ^13^, ^14^, ^15^, ^16^, ^17^, ^18^, ^19^, ^20^, ^21^, ^22^ There is a gap in the literature regarding the significance of nutritional markers and sarcopenia in the metastatic setting. In a cancer where the median overall survival for patients with advanced disease receiving palliative chemotherapy is only about 12 months, discovery of novel prognostic markers would be helpful to guide discussions with patients and families about treatment decisions and prognosis.

In this study, we aim to evaluate the prognostic utility of nutritional markers and sarcopenia in patients with de novo metastatic gastric and esophageal adenocarcinoma.

## 2 METHODS

Patients with de novo metastatic gastric or esophageal adenocarcinoma treated at the Princess Margaret Cancer Centre from 2010 to 2016 were identified from an institutional registry. Patients with documented anthropometric measures of height and weight as well as available pretreatment abdominal computed tomography (CT) imaging were included. Clinical data including baseline patient demographics, clinicopathologic characteristics, treatment, and follow‐up information was collected by a trained abstractor and verified by a second investigator. This study was approved by the University Health Network institutional review board.

### 2.1 Nutritional status assessment

The nutritional index (NRI) was calculated at time of diagnosis, as well as at the time of first restaging CT (8–12 weeks after start of chemotherapy) for patients with available data using the following formula: NRI = 1.519 × serum albumin (g/L) + 0.417 × (actual/estimated weight [kg] × 100). In our study, malnourished patients were defined as those having moderate to severe malnutrition, as defined by having an NRI < 97.5 according to previous studies.^2^, ^3^

### 2.2 Sarcopenia assessment

Pretreatment abdominal CT images were used for baseline sarcopenia measurements. The Slice‐O‐Matic software (Version 5.0; TomoVision) was used to assess the skeletal muscle index (SMI) at the third lumbar (L3) vertebra in cm^2^, which was then normalized by the square of the height (m^2^). Hounsfield units (HU) were used to identify skeletal muscle (threshold −29 to 150 HU). SMI cutoffs for sarcopenia were 34.4 cm^2^/m^2^ in females and 45.4 cm^2^/m^2^ in males based on previously established consensus.^23^ An outcome‐blinded radiologist (RA) repeated sarcopenia measurements for a randomly selected subset of patients with an intraclass correlation of 0.972 (95% confidence interval [CI] 0.938–0.987). For patients who received chemotherapy, SMI was recalculated at the time of first restaging CT (between 8 to 12 weeks after chemotherapy initiation). A clinically significant change in SMI was defined as an increase or decrease by ≥10%.

### 2.3 Statistical analysis

Time‐to‐event data were analyzed using the Kaplan–Meier method, with overall survival defined as the time from date of diagnosis to date of death. Cox proportional hazard (PH) models were used to identify prognostic factors for overall survival. All statistical analyses were performed in R version 3.5.2 (R Core Team 2018).

## 3 RESULTS

### 3.1 Patient characteristics

In total, 175 consecutive patients with de novo metastatic gastric or esophageal adenocarcinoma were included. Baseline characteristics and treatment information are shown in Table 1. At the time of diagnosis, the median age was 63 years, 69% were male, 79% had an ECOG performance status of 0–1, 62% had gastric and 38% had esophageal adenocarcinoma. Chemotherapy (with or without radiotherapy) was used in 71% of patients, palliative radiotherapy alone in 16%, and best supportive care alone in 13%.

**Table 1:** Baseline clinicopathologic characteristics.

Characteristic	N = 175 (%)
Age, median (range), years	63 (29–87)
Male	121 (69%)
Asian	32 (18%)
ECOG performance status	
0	42 (24%)
1	97 (55%)
≥2	36 (21%)
Primary tumor	
Gastric	108 (62%)
Esophageal	67 (38%)
Number of metastatic sites, mean (SD)	2.5 (1.1)
Sites of metastases^a^	
Lymph node only	18 (10%)
Visceral	130 (74%)
Bone	25 (14%)
Brain	2 (1%)
Treatment	
Chemotherapy^b^	124 (71%)
ECF(X)^c^	84
Platinum doublet	18
Cisplatin/5FU (capecitabine)/trastuzumab^d^	14
Other	8
Palliative radiotherapy alone	28 (16%)
Best supportive care alone	23 (13%)

^a^ Visceral metastases include solid organs excluding bone and brain. Bone refers to bone metastases with or without lymph node and/or other visceral metastases. Brain refers to brain metastases with or without lymph node and/or other visceral metastases.

^b^ Includes all patients who received chemotherapy, with or without palliative radiotherapy.

^c^ ECF = epirubicin, cisplatin, 5‐fluorouracil; X = capecitabine.

^d^ 5FU = 5‐fluorouracil.

### 3.2 Nutritional characteristics

Baseline nutritional characteristics are summarized in Table 2. In the 3 months preceding diagnosis, 70% of patients had a weight loss of ≥5%. The median BMI at diagnosis was 24.2 (range 15.7–39.8). Most patients had a normal BMI or were overweight by BMI, while only 5% of patients were classified as underweight using BMI. Using the NRI, 33% of patients were malnourished at baseline. Of the subset of malnourished patients, 80% had a history of weight loss, 60% were sarcopenic, and only 12% were underweight using BMI. About 68 patients (39%) were sarcopenic at baseline.

**Table 2:** Baseline nutritional characteristics.

Nutritional characteristic	N = 175 (%)
Weight loss ≥5%	122 (70%)
BMI (kg/m^2^)	
Underweight (<18.5)	9 (5%)
Normal (18.5–25)	92 (53%)
Overweight (>25)	74 (42%)
Nutritional Risk Index (NRI)	
Non‐malnourished (NRI≥97.5)	103 (67%)
Malnourished (NRI <97.5)	51 (33%)
Sarcopenia	68 (39%)

### 3.3 Treatment outcomes

**Figure 1:**
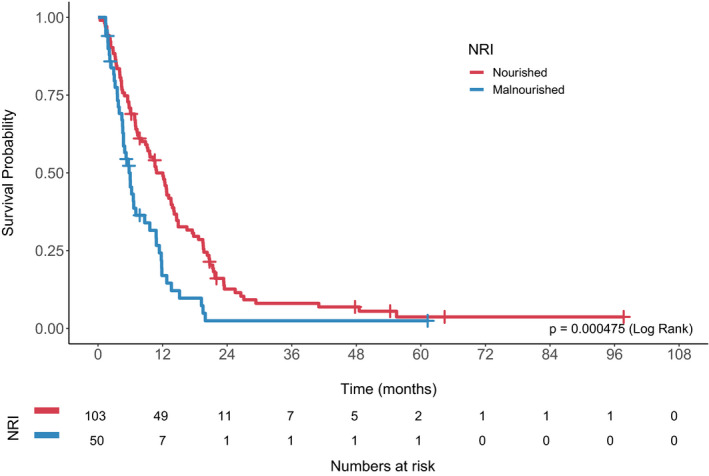
Overall survival by baseline Nutritional Risk Index (NRI).

**Figure 2:**
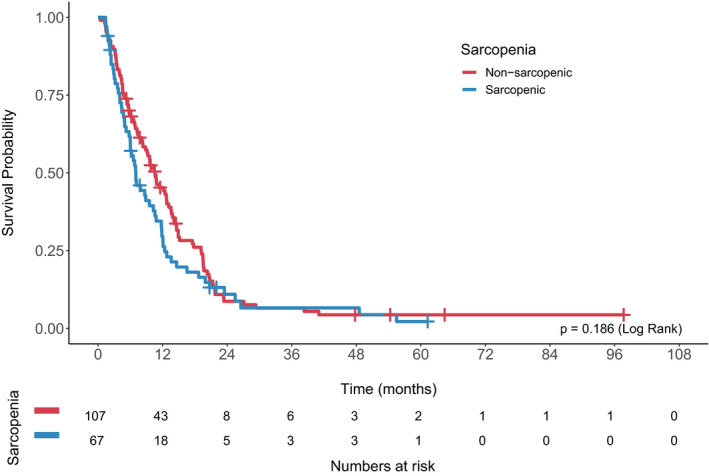
Overall survival by baseline sarcopenia status.

The median follow‐up time was 8.3 months (range 0.4–97.7). Median overall survival (OS) for the entire cohort was 9.3 months (95% CI 7.3–11.4). Overall survival was significantly worse in malnourished patients (median OS 5.8 vs. 10.9 months, log rank *p* = 0.000475; Figure 1). There was a trend toward decreased overall survival in sarcopenic patients, although this was not statistically significant (7.8 vs. 10.6 months, *p* = 0.186; Figure 2).

On univariable Cox PH analysis, ECOG (*p* < 0.001), number of metastatic sites (*p* = 0.029) and NRI (*p* < 0.001) were significant prognostic factors, while BMI (*p* = 0.57) and sarcopenia (*p* = 0.19) were not (Table 3). On multivariable analysis, ECOG (*p* < 0.001) and NRI (*p* = 0.025) remained significant as poor prognostic factors for OS (Table 3).

**Table 3:** Univariable and multivariable analysis for overall survival.

Covariate	Univariable		Multivariable	
	HR (95% CI)	*p*‐value	HR (95% CI)	*p*‐value
Age	1.05 (1–1.02)	0.066	1.01 (0.99–1.02)	0.44
Sex (male)	0.89 (0.63–1.25)	0.51	1.34 (0.87–2.06)	0.19
Race (non‐Asian)	0.76 (0.5–1.15)	0.2	0.83 (0.49–1.41)	0.5
Number of metastatic sites	1.17 (1.02–1.34)	**0.029**		
ECOG		**<0.001**		**<0.001**
0	Reference		Reference	
1	1.47 (0.99–2.17)	0.055	1.17 (0.74–1.84)	0.5
2	4.03 (2.23–7.26)	**<0.001**	2.64 (1.37–5.07)	**0.0037**
3–4	6.15 (3.34–11.3)	**<0.001**	9.28 (4.08–21.13)	**<0.001**
BMI < 18.5	1.22 (0.62–2.4)	0.57	0.66 (0.25–1.77)	0.41
Baseline NRI < 97.5	1.92 (1.32–2.78)	**<0.001**	1.77 (1.07–2.92)	**0.025**
Baseline Sarcopenia	1.24 (0.9–1.72)	0.19	0.97 (0.65–1.45)	0.88
NRI change		0.059		< **0.001**
Nourished → Nourished	Reference		Reference	
Malnourished → Nourished	1.94 (0.67–5.57)	0.22	2.45 (0.71–8.46)	0.16
Nourished → Malnourished	1.78 (0.93–3.42)	0.083	6.29 (2.53–15.65)	< **0.001**
Malnourished → Malnourished	2.17 (1.17–4.04)	**0.014**	7.11 (2.22–22.75)	< **0.001**
Change in SMI		0.63		0.098
Stable	Reference		Reference	
Increase 10%	0.67 (0.24–1.89)	0.44	0.29 (0.08–0.97)	0.045
Decrease 10%	1.09 (0.7–1.7)	0.7	1.19 (0.66–2.16)	0.56

### 3.4 Subset analysis of patients who received chemotherapy

**Figure 3:**
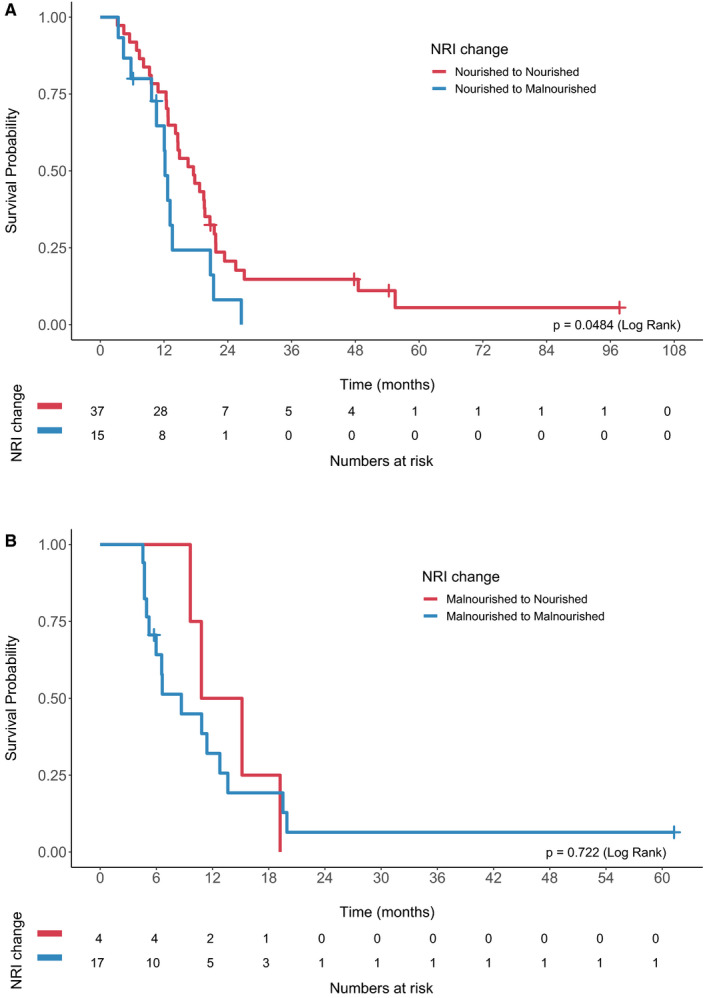
Overall survival of patients treated with chemotherapy stratified by change in NRI at time of first restaging CT scan. (A). Overall survival of nourished patients who became malnourished versus remained nourished. (B) Overall survival of malnourished patients who remained malnourished versus became nourished.

Of the included patients, 124 patients (71%) were treated with chemotherapy. At the time of first restaging CT scan, NRI could be recalculated in 74 patients. Overall survival stratified by change in NRI is shown in Figure 3. Patients who were not malnourished at baseline but became malnourished during treatment had significantly worse overall survival compared to those who maintained their nutritional status (12.2 vs. 17.5 months, log rank *p* = 0.0484; Figure 3A). Patients who were malnourished at baseline and remained malnourished had numerically worse survival compared to those who improved their nutritional status, although this was not statistically significant (8.7 vs. 13 months, log rank *p* = 0.722; Figure 3B). On multivariable analysis, change in NRI status was significantly associated with overall survival (Table 3).

**Figure 4:**
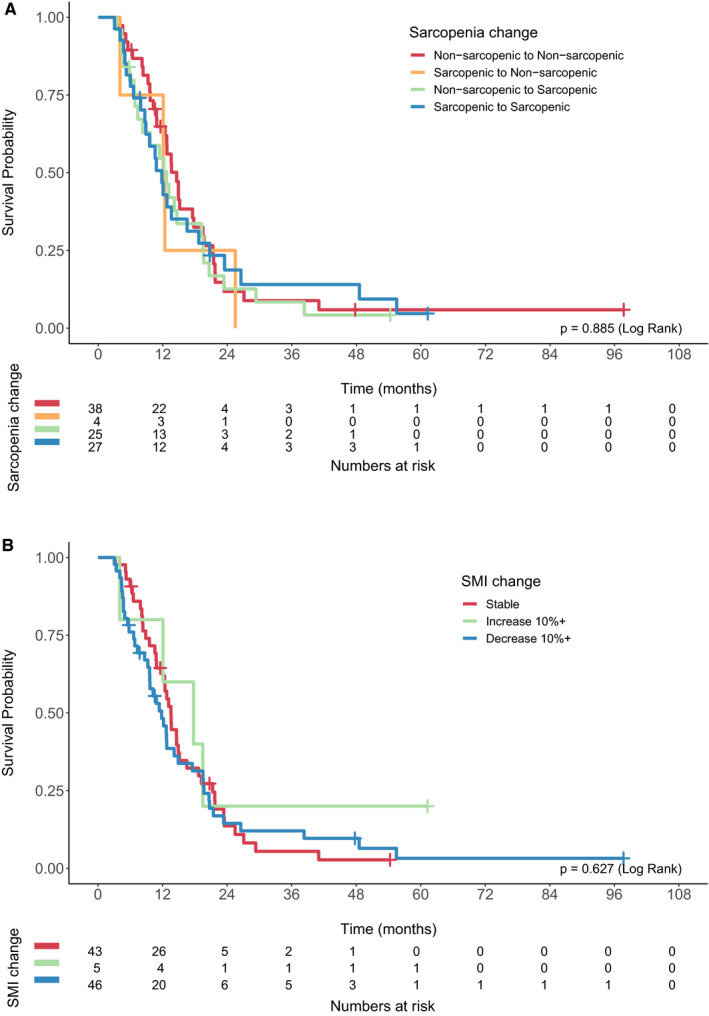
(A) Overall survival of patients treated with chemotherapy stratified by change in sarcopenia status. (B) Overall survival of patients treated with chemotherapy stratified by change in skeletal muscle index (SMI).

Restaging CT scans were available for sarcopenia analysis in 96 patients. There was no difference in survival between the groups based on change in sarcopenia status (log rank *p* = 0.885; Figure 4A). Overall survival stratified by change in SMI is shown in Figure 4B. The median overall survival for patients with stable versus increased versus decreased SMI were 13.6 months, 17.8 months, and 11.8 months, respectively (log rank *p* = 0.627).

## 4 DISCUSSION

This present study evaluated the prognostic significance of nutritional status and sarcopenia in patients with de novo metastatic gastric and esophageal adenocarcinoma, a group in which malnutrition and sarcopenia have not been extensively studied. We found that baseline NRI, as well as change in NRI during treatment, were significant prognostic markers for overall survival while sarcopenia status and skeletal muscle index were not.

Previous studies investigating NRI in esophageal cancer have centered on patients with localized disease undergoing curative‐intent treatment. Clavier and colleagues retrospectively analyzed 143 patients with localized esophageal cancer (113 squamous cell and 30 adenocarcinoma) treated with definitive concurrent chemoradiotherapy, and found on multivariable analysis that the NRI was an independent prognostic factor for overall survival.^3^ In a retrospective analysis of esophageal cancer patients included in the SCOPE1 clinical trial of chemoradiotherapy with or without cetuximab, Cox et al. reported that a baseline NRI < 100 was strongly associated with reduced overall survival.^4^ In the geriatric setting, two groups have studied the geriatric nutritional risk index (GNRI) in elderly patients with esophageal squamous cell carcinoma undergoing radiotherapy or definitive chemoradiotherapy.^5^, ^7^ In both studies, most patients had localized or locally advanced disease. Both groups found that the GNRI was independently associated with overall survival. Similarly, Kubo et al. retrospectively studied 240 esophageal squamous cell carcinoma patients who underwent esophagectomy, and found that the GNRI was an independent prognostic factor for overall survival.^6^ While these studies have consistently demonstrated that the NRI and GNRI are prognostic in esophageal cancer, particularly in those with squamous histology and localized disease, there is a paucity of literature in the metastatic setting, and in adenocarcinoma patients. To our knowledge, our study is the first to demonstrate the prognostic significance of NRI in a large cohort of de novo metastatic gastric and esophageal adenocarcinoma patients. Moreover, we are the first to show that the change in NRI over the course of treatment is prognostic for overall survival.

The existing sarcopenia literature in gastric and esophageal cancers has also focused on patients treated with curative intent, with inconsistent reports about the association between sarcopenia and prognosis. Multiple studies have found that sarcopenia is independently associated with poorer overall survival after curative‐intent resection or definitive chemoradiotherapy.^12^, ^13^, ^14^, ^15^, ^16^, ^17^, ^18^ However, almost an equal number of studies in the same time period and clinical context had the opposite finding that sarcopenia was not a significant prognostic factor for survival.^24^, ^25^, ^26^, ^27^, ^28^ To complicate interpretation, different methods were used to define sarcopenia in these studies. While most used the skeletal muscle index (SMI) at the L3 level on CT imaging, others used bioelectrical impedance, or incorporated functional measures of skeletal muscle weakness into the definition. Even among those who used the SMI at L3, different numerical reference ranges were used to define sarcopenia across the studies.

Most previous studies examined patients with localized or locally advanced disease undergoing definitive chemoradiotherapy or surgical resection. The few studies that exclusively looked at metastatic patients also had conflicting findings. In a cohort of 140 patients with advanced gastric cancer, Lee et al. reported that sarcopenic patients had significantly shorter overall survival.^21^ Conversely, Hayashi et al. found no significant difference in survival between patients with low versus normal skeletal muscle index.^22^ We also found that sarcopenia was not significantly associated with survival in our cohort of metastatic adenocarcinoma patients.

A few groups have reported that the loss of skeletal muscle mass and development of sarcopenia during disease course carries prognostic impact on oncologic outcomes, rather than just the presence of sarcopenia at baseline.^26^, ^29^, ^30^ Yoon et al. recently reported that although the presence of sarcopenia prior to neoadjuvant chemoradiotherapy and surgery was not prognostic, a 10% decrease in SMI during treatment was significantly associated with overall survival.^30^ Similarly, in the metastatic setting, Sugiyama et al. studied 231 patients with metastatic gastric cancer receiving platinum‐based chemotherapy, and found that though pretreatment sarcopenia was not associated with time to treatment failure or overall survival, muscle loss during treatment was associated with inferior survival outcomes.^20^ In our cohort, a decrease in skeletal muscle was not significantly associated with survival. In the few patients who demonstrated improved skeletal muscle index during treatment, there was a trend toward improved survival, although the small numbers in this subset prevent us from drawing definitive conclusions.

The limitations of our study include the retrospective design, limited number of patients in some groups of the subset analysis, and missing follow‐up imaging and laboratory data in some patients which prevented us from recalculating SMI and NRI at time of restaging. Notwithstanding these limitations, our study adds new insight to the existing literature, with several noteworthy findings.

Identification of malnutrition at diagnosis using the NRI was significantly associated with worse survival outcomes, and provided better prognostication compared to standard measures such as BMI. The NRI is a tool which can easily be used by clinicians, and may aid in treatment decision‐making and guide discussions of prognosis with patients and families by identifying those at high risk for poor outcomes. We also found that in patients who received chemotherapy, the change in NRI with treatment was associated with survival. In contrast to sarcopenia, which requires dedicated time and software for image analysis, the NRI is an objective measure which can be quickly calculated. In the clinical setting, use of NRI serially during treatment could provide useful prognostic information.

While NRI has been shown to be a useful poor prognostic marker, it is unclear if this is a risk factor that could be modifiable, or if this is simply a marker of aggressive disease. Prospective studies are needed to see if nutritional status can be improved with interventions in the clinical setting, and if doing so would improve outcomes in these patients.

## 5 CONCLUSION

This study is the first to demonstrate in a large cohort of de novo metastatic gastric and esophageal adenocarcinoma patients that pretreatment NRI as well as change in NRI during treatment course were significantly associated with poorer overall survival. NRI was superior to BMI alone as a nutritional prognostic marker. Further study is needed to determine whether these factors can be modified to improve prognosis in these patients.

## 6 ETHICS APPROVAL AND CONSENT TO PARTICIPATE

This study was approved by the Research Ethics Board of the University Health Network (approval number: 18‐5750.4). The study was performed in accordance with the Declaration of Helsinki.

## CONFLICT OF INTEREST

The authors declare no conflicts of interest.

## AUTHOR CONTRIBUTIONS

Study concept and design: Ma LX, Taylor K, Veit‐Haibach P, and Elimova E. Data collection and assembly: Ma LX, Taylor K, Anconina R, Suzuki C, and Bach Y. All authors participated in data analysis and interpretation, and manuscript revision. Final approval of manuscript: All authors.

## Data Availability

The data that support the findings of this study are available from the corresponding author upon reasonable request.
